# Self-wise, Other-wise, Streetwise (SOS) training: a novel intervention to reduce victimization in dual diagnosis psychiatric patients with substance use disorders: protocol for a randomized controlled trial

**DOI:** 10.1186/s12888-015-0652-1

**Published:** 2015-10-29

**Authors:** Marleen M. de Waal, Martijn J. Kikkert, Matthijs Blankers, Jack J. M. Dekker, Anna E. Goudriaan

**Affiliations:** Department of Research, Arkin Mental Health Care, Klaprozenweg 111, 1033 NN, Amsterdam, The Netherlands; Academic Medical Center, Department of Psychiatry, Amsterdam Institute for Addiction Research, University of Amsterdam, Amsterdam, The Netherlands; Trimbos Institute – Netherlands Institute of Mental Health and Addiction, Da Costakade 45, 3521 VS Utrecht, The Netherlands; Department of Clinical Psychology, Vrije Universiteit Amsterdam, Amsterdam, The Netherlands

**Keywords:** Victimization, Dual diagnosis, Substance use disorders, Severe mental illness, Intervention, Violence prevention, Emotion regulation, Social skills, Street skills, SOS training

## Abstract

**Background:**

Psychiatric patients are more likely to be victims of crime than others in the community. Dual diagnosis patients with comorbid psychiatric and substance use disorders are especially prone to victimization. Victimization is associated with substance abuse, more severe symptomatology and homelessness. There is a strong need for interventions to reduce victimization in this population. We developed the Self-wise, Other-wise, Streetwise (SOS) training to reduce victimization in patients with dual diagnosis.

**Methods/design:**

This study is a randomized controlled trial using a parallel group design to determine the effectiveness of adding the SOS training to care as usual. Patients with dual diagnosis (*N* = 250) will be allocated to either care as usual plus SOS training (*N* = 125) or care as usual only (*N* = 125) using computer-generated stratified block randomization. To compare effectiveness participants will be interviewed at baseline and 2, 8 and 14 months follow-up. The primary outcome measure is treatment response (yes/no), defined as either no victimization at 14 months follow-up or at least a 50 % reduction in incidents of victimization at 14 months follow-up compared to baseline assessment. Victimization is measured with the Safety Monitor, an adequate self-report instrument used by Statistics Netherlands to measure victimization on a large scale in the Netherlands. Outcome assessors are blind to treatment allocation. An economic evaluation will be performed alongside the randomized controlled trial and will take the societal perspective.

**Discussion:**

This study is the first randomized controlled trial to examine the effectiveness of an intervention that aims to reduce victimization in patients with dual diagnosis. If the intervention is effective it can be implemented in mental health care and contribute to the safety and well-being of patients.

**Trial registration:**

Dutch Trial Register (NTR): 4472, date of registration: 24-03-2014.

## Background

In contrast to what the general public assumes, psychiatric patients are more often victims of crime than perpetrators [1–3]. A systematic review on the prevalence of violent victimization of patients with severe mental illness (SMI) indicates that these patients are more likely to be violently victimized than other community members [2]. In addition to violent victimization, psychiatric patients are more often a victim of property crime [3]. Recent research in the Netherlands showed an annual prevalence rate of victimization in SMI outpatients of 47 % compared to 32 % in the general population [4]. Dual diagnosis patients with comorbid psychiatric and substance use disorders are especially prone to victimization [2, 5, 6]. These patients are approximately 1.3–5.4 times more likely to be violently victimized than psychiatric patients without substance use disorders [2, 5].

Victimization is associated with physical injury, psychological distress and impaired occupational functioning [7–9], but also with posttraumatic stress disorder, major depression, substance abuse and difficulties with emotion regulation and assertiveness [7, 9–13]. In SMI patients, victimization is associated with more severe symptomatology and poorer illness course [14–16], more substance abuse [14, 15, 17, 18], depression [18], homelessness [15, 17], violent behavior [19], offending [20] and interpersonal problems [21]. In substance-dependent patients, victimization is associated with psychological distress [22], worse psychiatric status, more psychiatric hospitalizations and outpatient treatment, worse general level of functioning [23], worse addiction illness-course [24] and offending [25].

Since most studies used cross-sectional designs, the exact mechanisms behind these associations remain unclear. It is however plausible that these problems associated with victimization, such as substance abuse, more severe symptomatology and homelessness, are consequences as well as predictors of victimization [12, 14, 24, 26–28]; This may cause a destructive vicious cycle of victimization, substance use and severe symptomatology. In line with this is the finding that an accurate predictor of victimization is having a history of victimization [26, 29–32]. One explanation of this victimization-revictimization link is that some are more prone to become a victim than others, due to personal characteristics, lifestyle and living environment [33, 34]. There is however evidence that revictimization is partly the result of changes following victimization, like emotion dysregulation, more substance use, offending, depression and posttraumatic stress disorder [12, 24, 32, 35].

Victimization of patients is an important concern not only because of its huge effect on mental and physical health, but also because of the significant societal and economic consequences of violence, due to increased burden on social services, healthcare and justice systems and productivity losses [36]. Reducing victimization in psychiatric patients may have long-lasting effects on personal well-being, the number of criminal offenses in society and economic costs. Consequently, there is a strong need for interventions to reduce victimization in psychiatric patients [2–4, 37–39]. Patients with dual diagnosis are the most vulnerable to victimization [2, 3, 5]. There is a lack of evidence-based treatment options for patients with substance use disorders co-occurring with severe mental illness [40] or posttraumatic stress disorder [41]. Therefore, we developed an intervention, the Self-wise, Other-wise, Streetwise (SOS) training, that aims to reduce vulnerability to victimization in patients with dual diagnosis.

SOS training focusses on the modifiable risk factors for victimization, which are subdivided into three different themes, represented in three training modules. The module *Self-wise* targets emotion regulation skills, the module *Other-wise* targets assertiveness and conflict resolution skills and the module *Streetwise* targets knowledge and skills to enhance personal safety and reduce vulnerability. As no evidence-based interventions are available so far, SOS training is based on evidence based treatment programs in other patient groups that target these skills [42–46]. SOS training is adapted to the needs of patients with substance use disorders co-occurring with any other mental disorders and can therefore be widely used in clinical practice.

By adding SOS training to care as usual (CAU) we expect patients to improve on psychopathology, emotion regulation skills, interpersonal problems and self-esteem. Besides, adding SOS training to CAU is expected to result in a decrease in substance use. We expect that these improvements will result in a reduction of victimization.

### Aims of the trial

This study aims to investigate the effectiveness of SOS training in reducing victimization of patients with dual diagnosis.

The primary research question is:

1. Does adding SOS training to CAU result in a larger reduction of victimization compared to CAU alone?

Secondary research questions are:

2. Does adding SOS training to CAU result in a larger improvement on secondary outcome measures compared to CAU alone?
3. How do costs and effects of SOS training + CAU compare to CAU from a societal perspective?

## Methods/design

### Design

This study is a single-blind two-arm randomized controlled trial using a parallel group design to determine the effectiveness of adding SOS training to CAU. Participants will be interviewed at baseline and 2, 8 and 14 months follow-up. After baseline assessment, participants will be randomly allocated to either CAU + SOS training or CAU.

### Participants

Participants will be recruited in an addiction-psychiatry clinic and an addiction-psychiatry outpatient care facility in Amsterdam. Inclusion criteria are (1) 18 years of age or older; (2) substance dependence or substance abuse (involving alcohol and/or illegal drugs, including cannabis) according to Diagnostic and Statistical Manual of Mental Disorders-IV (DSM-IV) criteria; and (3) at least one other mental disorder on DSM-IV Axis I or II. Patients are excluded from the study if they (1) do not have sufficient understanding of the Dutch language; or (2) are not eligible for group therapy according to their case manager, due to for instance severe anti-social or psychopathic traits; (3) are not willing to provide informed consent. Based on a priori power analysis, we aim to include 250 (2x125) participants in this study.

### Interventions

#### Self-wise, Other-wise, Streetwise (SOS) training

The experimental add-on intervention, SOS training, is a 12 session group-based training [47]. Sessions take place twice a week and take 75 min. Each session utilizes learning techniques such as role playing, visual material, group discussions and sharing experiences. The trainers create a safe and respectful environment. Participants can give each other tokens of appreciation during every session, in order to reinforce desirable behaviour. These tokens are cards with coloured stars. By using the tokens, participants learn to reinforce and support each other. Furthermore, the tokens stimulate the participants to listen to and interact with each other. The training comprises 3 modules, *Self-wise, Other-wise* and *Streetwise.* Each module consists of 4 sessions. There is no specific order in which participants follow these modules.

The module *Self-wise* involves a transdiagnostic emotion regulation skills training. Emotion regulation refers to the ability to monitor, evaluate and modify emotional reactions to accomplish one’s goals [48]. Emotion dysregulation is a predictor of victimization [12, 49–51]. Lack of emotional awareness, for instance the inability to recognize and interpret fear cues, impairs risk perception [12, 52]. Poor risk perception increases the risk for victimization [49, 52]. Emotion dysregulation, especially impulsivity, also increases the likelihood of entering a risky situation and makes it more difficult to leave or adequately cope with a dangerous situation [12, 52]. Even small increases in emotion regulation can impact victimization risk substantially [12]. Since no evidence-based emotion regulation skills training is available for our target group, the content of *Self-wise* is inspired by elements of affect regulation therapy for major depressive disorder [42], anger management training for people with intellectual disabilities [43], skills training in affect and interpersonal regulation for patients with posttraumatic stress disorder related to childhood abuse [44] and the system training for emotional predictability and problem solving for patients with borderline personality disorder [45]. *Self-wise* is a new, simplified emotion regulation skills training suitable for patients with dual diagnosis, based on the principles of these interventions.

The module *Other-wise* involves a conflict resolution skills training. Offending, violent behaviour, interpersonal problems and lack of assertiveness increase the risk for victimization [13, 19–21, 25]. Aggressive responses to provocation can easily lead to an escalation of violence. On the other hand, non-assertive responses can provoke manipulation and exploitation. The content of *Other-wise* is inspired by social skills training for patients with schizophrenia [46]. In this module patients compose a list of important skills for preventing and resolving conflicts, which are practiced in role-playing exercises categorized by theme. Examples of these themes are: Responding to Frustrating Situations, Handling Bad News and Leaving Unsafe Situations. The specific role-playing exercises within these themes are based on input from dual diagnosis patients and mental health professionals working with these patients.

The module *Streetwise* involves a street skills training. It has been suggested that teaching patients about changeable factors that contribute to their risk of victimization may be effective in reducing victimization [37, 53–55]. *Streetwise* builds on the suggested strategies that make patients reflect on the safety of their environment and their own contribution to safety. An important aspect of *Streetwise* is safety regarding drug related behaviour and contact with drug dealers. All knowledge and skills are transferred in a playful, stimulating manner, using role-play and creative exercises.

#### CAU: Care as usual

Most participants will suffer from chronic mental health problems. Hence, CAU is an ongoing treatment process. CAU usually consists of pharmacotherapy in combination with a form of case management, for instance assertive community treatment [56]. In addition, since SOS training will be imbedded in addiction-psychiatry treatment programs, when enrolling in this study most participants will attend some form of psychosocial therapy. Depending on the participants’ motivation, capacities and needs this may include: cognitive behavioural therapy [57], motivational interviewing [58], substance abuse management training [59], social skills training, psychomotor therapy and mindfulness. The type and amount of care received by participants will be recorded.

#### Therapists

SOS training will be implemented in the treatment programs of an addiction-psychiatry dual diagnosis clinic and an addiction-psychiatry outpatient care facility of Arkin Mental Health Care, division Mentrum. Arkin is an Amsterdam-based public mental health care treatment facility. Eight employees are selected to be trained to deliver the intervention. These SOS-trainers work in various positions, for instance as psychologist, nurse or social worker. All have ample experience in working with psychiatric patients with substance use disorders and are motivated to implement this new intervention. The SOS-trainers will follow a detailed training manual which describes each training session. Each session will be led by an SOS-trainer accompanied by an SOS-co-trainer.

#### Therapist education

The education for SOS-trainers includes: studying the SOS training manual independently and two 3-h group training sessions, which will be provided by one of the researchers. The first group training session consists of: the theoretical framework of SOS training, an explanation of the exercises in module Streetwise and practising these exercises. The second group training session consists of: an explanation of the exercises in module Self-wise and Other-wise and practising these exercises. The researcher who provides the education for SOS-trainers will be present at the delivery of the first 12 sessions SOS training on both locations, to provide additional feedback to the SOS-trainers after each session. Thereafter, this researcher will attend an SOS training session at least once every month on both locations. After 6 months, an individual evaluation will be held with every SOS-trainer.

### Procedure

#### Recruitment and consent

Patients will be recruited in an addiction-psychiatry dual diagnosis clinic and an addiction-psychiatry outpatient care facility. All patients who fit the inclusion criteria, will be informed and invited by their mental health care facility. Participants will receive a participation compensation of 15 Euros per assessment for T0, T1 and T2 and 30 Euros for T3. Written informed consent will be provided before the first assessment takes place.

### Randomization and treatment allocation

Participants will be allocated to CAU + SOS training or CAU by an independent researcher using a computer-generated block randomization schedule. Randomization will be stratified by two prognostic factors: treatment centre (clinic/outpatient care) and victimization in the year prior to baseline (yes/no). Block size varies randomly. To prevent selection bias, the research coordinator and outcome assessors are denied access to the randomization schedule and are blind to block size and order.

#### Trial flow

**Fig. 1:**
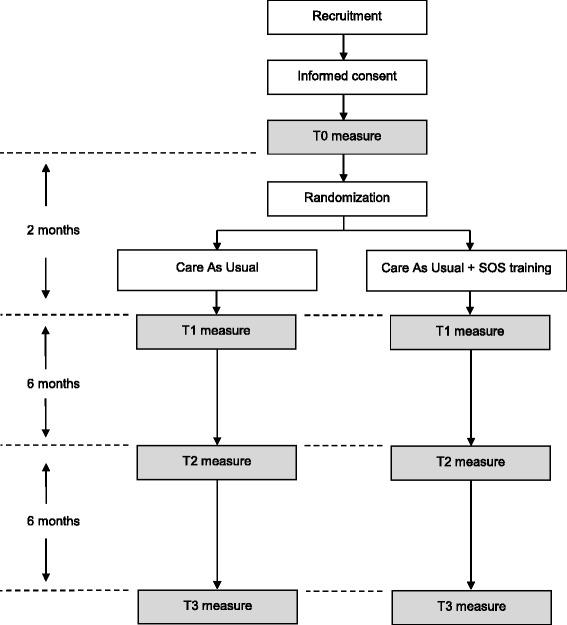
Flow chart of the study design

Figure 1 provides an overview of the trial flow diagram. After informed consent is provided and baseline assessment (T0) has taken place, participants will be randomized and allocated to either CAU + SOS training or continue to receive CAU. Follow-up assessments will take place 2, 8 and 14 months after baseline assessment. For participants allocated to trial arm CAU + SOS training this will correspond to immediately, 6 months and 12 months after the completion of SOS training.

### Measurements

Table 1 provides an overview of the measurement instruments. All instruments will be administered in the form of an interview, by a junior researcher (MSc in Psychology) or master student (holding a BSc) in clinical psychology. To minimize bias, the junior researcher will train the master student in carefully collecting data and interviewing participants. This training will start with two days consisting of explanation and role plays. After that, the master student will watch the junior researcher administer assessments. Finally, the junior researcher will supervise the first assessments administered by the master student and give feedback.

**Table 1:** Overview of measurement instruments

Measurement instrument	T0	T1	T2	T3^a^
Safety Monitor	x			x
Questions regarding likelihood and controllability of victimization	x	x	x	x
Timeline Followback	x	x	x	x
Alcohol Use Disorder Identification Test	x		x	x
Drug Use Disorder Identification Test	x		x	x
Brief Psychiatric Rating Scale	x	x	x	x
Kessler psychological distress scale	x	x	x	x
Difficulties in Emotion Regulation Scale	x	x	x	x
Dimensions of Anger Reactions	x	x	x	x
Inventory of Interpersonal Problems	x	x	x	x
Self Esteem Rating Scale	x	x	x	x
EuroQol 5D	x	x	x	x
Manchester Short Assessment of Quality of Life	x	x	x	x
Trimbos questionnaire on Costs associated with Psychiatric illness		x	x	x
Jellinek PTSD Screening Questionnaire	x			x
Posttraumatic Diagnostic Scale	x			x
Mini-Mental State Examination	x			
Client Satisfaction Questionnaire		x	x	x

^a^ *T0* baseline, *T1* 2 months follow-up, *T2* 8 months follow-up, *T3* 14 months follow-up

Both outcome assessors will be blind to the trial arm to which the participant is allocated after randomization. To verify blinding, after each follow-up assessment the assessor will record to which trial arm he/she thinks the participant is allocated. Furthermore, the assessor will record his/her confidence regarding these thoughts on allocation.

### Primary outcome measure: victimization

Victimization will be measured with the Dutch version of the Safety Monitor (in Dutch: Veiligheidsmonitor, VM section 4), developed by the Dutch Ministry of Security and Justice [60]. The Safety Monitor is an instrument used by Statistics Netherlands (CBS) to measure victimization on a large scale (almost 80 thousand cases a year). It is an adequate self-report instrument that strongly resembles the International Crime Victimization Survey (ICVS) [61]. Section 4 of the Safety Monitor assesses victimization of 11 different crimes, subdivided in three categories:

- violent crimes, consisting of: sexual crimes, threats and assaults;
- property crimes, consisting of: burglary, theft from car, car theft, motor vehicle theft, bicycle theft, robbery and theft of other property;
- vandalism;

The Safety Monitor examines whether participants experienced each specific crime in the last 5 years. If so, they are asked whether they experienced that crime in the last 12 months. For each crime reported, participants are asked how frequently they experienced that crime in the last 12 months.

The primary outcome measure is treatment response (yes/no), defined as either no victimization at T3 or at least a 50 % reduction in incidents of victimization at T3 compared to T0. Since there are no previous randomized controlled trials available that aimed to reduce victimization in this target group, our treatment response criteria are based on experts opinions. Mental health care professionals working with dual diagnosis patients indicated a 50 % reduction in incidents of victimization as a clinically relevant and achievable goal.

### Key secondary outcome measures

#### Violent victimization and property victimization

The Safety Monitor subcategories violent victimization and property victimization will be examined separately.

#### Substance use (disorders)

Substance use in the past 90 days will be measured with the Timeline Followback (TLFB) [62], which has good reported reliability and validity in dual diagnosis populations [63, 64]. Using calendars, beginning on the day of assessment and working backwards, participants reconstruct daily substance use.

The Alcohol Use Disorder Identification Test (AUDIT) [65], developed by the World Health Organization (WHO) will be included to measure alcohol consumption burden. The AUDIT has shown good reliability and validity in various target groups [66] including individuals with severe mental illness [66–68]. The Drug Use Disorder Identification Test (DUDIT) [69], developed as a parallel instrument to the AUDIT, will be included to measure drug consumption burden. The DUDIT has shown good reliability and validity in individuals with psychosis [70] and in substance abusers in various treatment settings [69, 71].

#### Psychopathology

Psychopathology will be measured with the Brief Psychiatric Rating Scale-Expanded (BPRS-E) [72, 73], which evaluates a broad range of psychiatric symptoms. The BPRS-E is often used as measure of symptom outcome in psychiatric populations [74]. The clinician-administered instrument is sensitive to change in symptom severity, is valid [74, 75] and reliable [74, 76], also in patients with dual diagnosis [77].

#### Emotion dysregulation

Emotion dysregulation will be measured with the Difficulties in Emotion Regulation Scale (DERS) [78]. The DERS evaluates clinically relevant emotion regulation difficulties across multiple domains, represented in six subscales: non-acceptance of emotional responses, difficulty engaging in goal-directed behaviour, impulse control difficulties, lack of emotional awareness, limited access to emotion regulation strategies and lack of emotional clarity. The DERS is used extensively as outcome variable in clinical research and has high internal consistency and good test-retest reliability and construct validity [78, 79], also in patients with SMI [80].

### Other secondary outcome measures

Other secondary outcome measures are:

- interpersonal functioning as measured with the Inventory of Interpersonal Problems (IIP-32) [81],
- self-esteem as measured with the Self Esteem Rating Scale (SERS-SF 20) [82],
- anger disposition as measured with the Dimensions of Anger Reactions (DAR) [83],
- psychological distress as measured with the Kessler psychological distress scale (K10) [84, 85],
- quality of life as measured with the EuroQol 5D (EQ-5D-5 L) [86] and the Manchester Short Assessment of Quality of Life (MANSA) [87],
- healthcare costs and productivity losses/gains as measured with the Trimbos/iMTA questionnaire on Costs associated with Psychiatric illness (TiC-P) [88].

### Other variables of interest

#### General patient characteristics

General demographic characteristics such as age, gender, marital status and educational level will be collected at baseline. Furthermore, current DSM-IV diagnosis, total days of hospitalisation, all-cause mortality, Global Assessment of Functioning (GAF) and mental healthcare received by participants will be extracted from the electronic patient record.

#### Context of victimization

For all crimes reported, context information on victimization will be examined with the Safety Monitor. Each crime reported will be followed by an exploration of the most recent incident. The Safety Monitor contains questions on when and where the incident happened, who the perpetrator was and whether the police was informed. For perpetrator we added the answer options ‘friend’, ‘drug dealer’, ‘other patient’ and 'health care worker’ in addition to the existing options.

In addition to the crimes described in the section ‘primary outcome measure’, four new types of crime that were added in the most recent version of the Safety Monitor will be examined. These new types of crime are: identity fraud, sales fraud, hacking and cyberbullying.

To obtain more detailed information on victimization we extended the Safety Monitor with extra questions. For sexual crimes, threats, assaults and robbery we examine what led to the incident, whether the patient used any substances at the time of the incident and, if relevant, why the participant did not inform the police.

#### Likelihood and controllability of victimization

For each crime assessed with the Safety Monitor, questions are added that examine the perceived likelihood and controllability of becoming a victim of this crime. These questions are based on a risk perception study by Jackson [89]. Participants are asked: ‘to what extent do you feel able to control whether or not you become a victim of the following?’ and ‘how likely do you think it is that you will fall victim of each of the following during the next twelve months?’. To obtain conceptual equivalence, three of the researchers independently translated the questions to Dutch and then formed a panel to resolve discrepancies in these forward translations. Subsequently, an independent translator translated the questions back to English. We discussed discrepancies with this translator until we reached consensus. Finally, we successfully pre-tested the questions on 10 patients from our study population.

#### Perpetration

Perpetration will be measured with the extended version of the Safety Monitor. For each crime, participants are asked if they have committed that crime in the last 5 years. If so, participants are asked if and how often they permitted that crime in the last 12 months. Prior to the assessment of perpetration, participants will be reminded of the confidentiality of the assessment.

#### Within-day relationship victimization and substance use

The within-day relationship between victimization and substance use will be examined with the TLFB [62]. First, substance use will be administered with the TLFB, using calendars, as described in the section ‘secondary outcome measures’. Thereafter participants will reconstruct victimization in the past 90 days, following the same procedure, beginning on the day of assessment and working backwards. For each day on which both victimization and substance use are reported, the within-day process will be examined. Participants will be asked to indicate whether they used substances before being victimized, after being victimized, or both. The TLFB has previously been used to assess partner violence and victimization and their within-day relation to substance use [90–93].

#### Post-traumatic stress disorder

The Jellinek PTSD Screening Questionnaire [94] will be included to screen for post-traumatic stress disorder symptoms. If a participant tests positive, post-traumatic stress disorder symptoms are measured using the Posttraumatic Diagnostic Scale (PDS) [95].

#### Cognitive impairment

The Mini-Mental State Examination (MMSE) will be included to screen for cognitive impairment.

#### Client satisfaction with treatment services

The Client Satisfaction Questionnaire (CSQ-8) [96, 97] will be included to measure participants’ satisfaction with their mental health treatment services.

### Observation scales

For each participant in the clinic, two observation scales will be independently administered by two out of eight nurses, who are not otherwise involved in the SOS training or research. Social functioning will be measured with the Personal and Social Performance scale (PSP) [98]. Aggressive and social behaviour will be measured with the Observation Scale for Aggressive Behaviour (OSAB) [99], which consists of the subscales: irritation/anger, anxiety/gloominess, aggressive behaviour, antecedent (to aggressive behaviour), sanction (for aggressive behaviour) and social behaviour. Due to practical reasons, the observation scales will only be administered for hospitalized participants.

### Evaluation of treatment

After each training session, the SOS-trainers will fill in a session evaluation form in which they note to what extent they followed the instructions in the SOS training manual (scale 1–10) and which participants were present, including a mark (scale 1–10) for effort.

After completing each module of SOS training, participants randomized to CAU + SOS training will fill in a questionnaire to evaluate that module. Each evaluation contains the questions: ‘how much fun was this module to you?’ and ‘how helpful was this module to you?’ (both scale 0–10). Subsequently, participants rate three module-specific skills on a scale from 0 to 100, with 0 being ‘I did not improve on this at all’ and 100 being ‘I improved on this a lot’. Finally, participants will rate how much they improved on self-confidence and on controllability of becoming a victim of crime, due to following this module (scale from 0 to 100).

### Data analysis

#### Sample size calculation

A priori sample size calculations were performed using G*power 3.1.9.2. We are the first to explore effects of an intervention to reduce victimization in this target group, which makes it impossible to provide an exact estimate of effect size for the main outcome measure in this sample. In order to be able to detect a 20 % difference in treatment response between the two trial arms (50 % treatment response in SOS training + CAU versus 30 % treatment response in CAU) with α = .05 and power = .80, the total sample size should be at least 186 (N CAU + SOS training = 93, N CAU = 93). Since a drop-out of 25 % can be expected in this target group we aim to include 250 participants.

#### Effectiveness

Primary data analyses will be performed in accordance with the intention-to-treat paradigm. In addition, per protocol analyses will be conducted. Missing data will be addressed using multiple imputation. Statistical significance will be set at α < .05, based on two-sided tests. The effect of treatment in terms of the primary and secondary outcome variables will be analysed with generalized linear mixed model regression analysis (GLMM) with adequate link functions, taking into account distributional characteristics of the data.

#### Costs

We will consider four types of costs: (1) the costs of offering the intervention (SOS training + CAU or CAU), (2) costs stemming from general health care uptake besides SOS training + CAU or CAU, including the costs of medication, (3) patients' out-of-pocket expenses (eg traveling costs, leisure time spent on receiving care), (4) costs stemming from productivity losses due to absenteeism or reduced efficiency while at work (presenteeism). The first two types of costs are also known as the direct medical costs and these will be based on the full economic costs of offering the interventions. Here, we will use the pertinent Dutch guideline for economic evaluation [100], and rely on the standard cost prices reported therein. Productivity losses will be based on the gender- and age-specific labour costs. Data on resource use (health care uptake) and productivity losses will be collected with the widely used TiC-P [88].

#### Effects

As effect measure, the 5-level variant of the 5-dimensional EuroQol instrument (EQ-5D-5 L) [86] will be used to compute health gains expressed in quality adjusted life years (QALYs). Health utilities are a major feature of the EQ-5D instrument. As the EQ-5D-5 L is relatively recent update of the 3-level EQ-5D, studies that directly elicit preferences from general population samples to derive value sets to calculate the EQ-5D-5 L health utilities are under development in a number of countries, including the Netherlands [101]. In the interim, the EuroQol Group coordinated a study that administered both the 3-level and 5-level versions of the EQ-5D, in order to develop a “crosswalk” between the EQ-5D-3 L value sets and the new EQ-5D-5 L descriptive system, resulting in crosswalk value sets for the EQ-5D-5 L [102]. It is expected that the new utilities for the EQ-5D-5 L will be available when we will perform the calculations for the economic evaluation; otherwise we will use the crosswalk from the EuroQol Group to calculate health utilities.

#### Cost-effectiveness calculations

The economic evaluation will be conducted alongside the randomized trial, taking into account the CHEERS statement [103] and the 2015 ISPOR good research practices task force report on cost-effectiveness analysis alongside clinical trials [104]. Technically, the economic evaluation referred to as cost-effectiveness analysis in the following section will be a cost-utility analysis as health utilities are used as the effect measure.

Using the area under the curve (AUC) method, the periods between the measurement waves will be weighted by the utility of the health state in that period. This allows the computation of quality adjusted life years (QALYs) over the entire trial period. In similar vein, cumulative costs over the entire follow-up period will be obtained from the cost estimates at the various measurement waves. The cost-utility evaluation will be performed in line with suggestions by Drummond et al. (2005) [105], i.e. in agreement with the intention-to-treat principle, with missing data addressed using imputation.

The incremental cost-effectiveness ratio (ICER) will be calculated as follows: ICER = (C1-C2)/(E1-E2), where C stands for costs, E for effects, and subscripts 1 and 2 refer to the two trial arms (SOS training + CAU and CAU, respectively). Confidence intervals around the ICER will be calculated using a non-parametric bootstrap approach: 5,000 non-parametric bootstrapped samples will be extracted from the original dataset. For each of these bootstrapped samples, the incremental costs, incremental effects, and the incremental cost-effectiveness ratio (ICER) will be calculated. The resulting 5,000 ICERs will be used for further calculations and will be plotted on a cost-effectiveness plane. In addition, cost-effectiveness acceptability curves (CEACs) will be constructed. One-way sensitivity analyses directed at uncertainty in the main cost drivers will be performed to gauge the robustness of our findings. In addition, a sensitivity analysis in which a covariate-adjusted CEAC curve is constructed will be conducted using net benefit regression methods [106, 107].

## Ethical approval

This RCT has been reviewed and approved by the ethics committee of the Academic Medical Center of the University of Amsterdam, Amsterdam, The Netherlands and will be conducted in accordance with the Declaration of Helsinki [108].

## Discussion

Patients with comorbid mental and substance use disorders are vulnerable to victimization [1–5]. There is a strong need for interventions that reduce victimization of patients [2–4, 37, 53]. This randomized clinical trial is the first to test the effectiveness of an intervention that aims to reduce vulnerability for victimization in patients with dual diagnosis. We will test whether adding the Self-wise, Other-wise, Streetwise (SOS) training to care as usual results in a reduction of victimization. Besides, we will examine improvement on secondary outcome measures and cost-effectiveness.

Previous studies testing the effectiveness of psychosocial interventions in similar target groups have been hampered by small samples, poor experimental design, short follow-up periods and high loss to treatment and loss to follow-up [40]. Major strengths of this study are the follow-up period of 14 months, the relatively large sample size of 250 individuals and the large battery of validated and clinically relevant outcome measures. Furthermore, those assessing outcomes will be blind to treatment allocation. Our main challenges in this study are expected to be treatment adherence and preventing drop-out. We will work closely together with patients’ mental health care professionals to prevent drop-out as much as possible. Moreover, participants will receive monetary compensation for completing follow-up assessments. Finally, missing data will be addressed using multiple imputation.

If SOS training is effective in reducing vulnerability for victimization it can be implemented in mental health care and contribute to the safety and well-being of patients.
